# Disturbances in Central Sensitization Are Associated with Disease Severity and Alterations in Gene Expression Measured in the Peripheral Blood Mononuclear Cells of Patients with Rheumatoid Arthritis

**DOI:** 10.3390/ijms27062872

**Published:** 2026-03-22

**Authors:** Elena Tchetina, Alena Potapova, Angele Vienozinskaite, Svetlana Glukhova, Maria Cherkasova, Ekaterina Filatova, Andrey Karateev, Aleksandr Lila

**Affiliations:** 1 Immunology and Molecular Biology Laboratory, Nasonova Research Institute of Rheumatology, Kashirskoe Shosse 34A, 115522 Moscow, Russia; angelina_vienozinskaite@hotmail.com (A.V.); maricherk@yandex.ru (M.C.); amlila@mail.ru (A.L.); 2 Laboratory of Pathophysiology of Pain and Polymorphism of Rheumatic Diseases, Nasonova Research Institute of Rheumatology, Kashirskoe Shosse 34A, 115522 Moscow, Russia; dr.aspotapova@mail.ru (A.P.); es-filatova@mail.ru (E.F.); aekarat@yandex.ru (A.K.); 3 Statistics Department, Nasonova Research Institute of Rheumatology, Kashirskoe Shosse 34A, 115522 Moscow, Russia; sveglukhova@yandex.ru

**Keywords:** rheumatoid arthritis, central sensitization, gene expression, metabolism, PBMCs

## Abstract

Rheumatoid arthritis (RA) is a chronic autoimmune rheumatic disease of unknown etiolgy, characterized by erosive polyarthritis that leads to joint destruction and systemic inflammatory lesions in internal organs. Pain is a primary symptom of RA and a major contributor to psychological disturbances, which influence patients’ subjective evaluation of their condition. These psychological issues may stem from disruptions in central pain regulation mechanisms, such as central sensitization (CS), which can also affect central metabolic processes. The objective was to investigate how the severity of central sensitization, measured by the Central Sensitization Inventory (CSI) questionnaire (Part 1), impacts clinical and neuropsychiatric parameters, as well as the expression of genes related to inflammation, tissue destruction, carbohydrate metabolism, and fatty acid metabolism in peripheral blood mononuclear cells (PBMCs) in patients with RA. Methods involved collecting blood samples from 59 RA patients (mean age 52.0 years). Clinical status was assessed using the DAS28 index and serum levels of CRP, ASPA, and RF. Neuropsychiatric parameters were evaluated through questionnaires measuring CS severity score (CSI), pain intensity (VAS, BPI), neuropathic pain (PainDETECT), anxiety and depression (HADS), fatigue (FSS, FACIT-F), fibromyalgia symptoms (FIRST), and pain catastrophizing. Protein expression in PBMCs was measured by ELISA, while gene expression was analyzed using quantitative real-time RT-PCR. All patients exhibited moderate to high disease activity. Participants were divided into four subgroups according to their CSI scores: subclinical (0–29 points), mild (30–39 points), moderate (40–49 points), and severe/extreme (50–100 points). Higher CSI scores correlated with significant increases in neuropsychiatric symptoms and a notable decrease in vitality. However, clinical parameters showed no significant differences among the subgroups. Gene expression analysis revealed upregulation of genes involved in the pentose phosphate pathway (G6PD), antioxidant defense (SOD1), fatty acid metabolism (FASN, CPT1B), apoptosis (CASP3), and tissue destruction and hypernociception (MMP-9) compared to healthy controls. The pro-inflammatory cytokine IL-1β expression was comparable to controls, while TNFα expression was elevated only in patients with severe/extreme CS scores. These findings suggest that CS-related disturbances may contribute to increased disease severity in RA, even in patients receiving active antirheumatic treatment. At the cellular level, disease severity appears linked to dysregulated expression of genes governing central metabolic processes, despite low expression of pro-inflammatory cytokine genes.

## 1. Introduction

Rheumatoid arthritis (RA) is an autoimmune disease marked by inflammation, pain, stiffness, and gradual destruction of joints and organs. Because inflammation is central to RA, the primary goals of antirheumatic therapy are to reduce joint inflammation and pain, improve joint function, and prevent tissue damage [1]. The prevalence of RA among adults varies widely, ranging from 0.3% to 4.2% depending on the population [2]. Pain is a defining feature of RA. It often appears before other clinical signs, contributes to psychological stress, disrupts sleep, and limits patients’ daily activities [3]. Pain also heavily influences patients’ subjective evaluation of disease severity, sometimes leading to a mismatch between physician-assessed inflammation and patient-reported pain—this discrepancy is seen in about 60% of RA patients [4]. During acute synovitis, pain intensity usually correlates with inflammation severity. However, pain can persist despite effective therapy, including clinical remission as measured by the DAS28 score [5,6]. This indicates that pain in RA is not solely due to joint pathology but reflects complex interactions involving peripheral, spinal, and supraspinal pain pathways. Specifically, it depends on both the direct activation of peripheral pain receptors and alterations in neuronal sensitivity along the entire pain signaling pathway [7].

The synovium and joint capsule contain peripheral afferent fibers from the dorsal root ganglion, which include neurons responsible for both mechanosensory and nociceptive functions [8]. These fibers can be activated by damage to the bone or articular cartilage [9]. Consequently, persistent pain in the absence of inflammation or local joint destruction—which occurs in 41% of patients with RA—is believed to be linked to disturbances in central pain regulatory mechanisms. These disturbances include dysfunction of descending inhibitory and facilitatory pathways, as well as central sensitization at the spinal cord level [10]. Central sensitization (CS) is a condition where the central nervous system (CNS) becomes hypersensitive to sensory input, leading to increased pain perception [11]. Chronic pain states associated with CS can trigger metabolic changes in CNS cells. For instance, gene expression alterations related to glycolysis and oxidative phosphorylation can reprogram glial cell metabolism [12]. Additionally, stress and chronic pain can affect hormone levels, such as cortisol, which in turn influence expression of genes involved in glucose and lipid metabolism [13]. Furthermore, CS disturbances may be connected to mitochondrial dysfunction, affecting energy production, mitochondrial biogenesis, and overall function—factors that can contribute to the persistence of pain in RA [14].

It has been previously demonstrated that patients with RA showing features of central sensitization (CS) experience higher VAS pain scores and greater fatigue. They are more often diagnosed with neuropathic pain descriptors, depression, anxiety, and fibromyalgia, and report significantly lower satisfaction with their condition despite receiving active antirheumatic therapy [15]. Although recent progress has been made in understanding neuroimmune communication and the impact of peripheral immune activation on neural circuits in RA, the exact mechanisms underlying CS-related pain remain unclear. Diagnosis still relies on a combination of clinical parameters, expert assessment, and qualitative sensory testing [16]. Therefore, investigating the mechanisms of CS is crucial for identifying new therapeutic strategies to alleviate pain and prevent its progression in RA patients. Pathological processes in RA trigger autoimmune responses along with disruptions in essential metabolic functions that influence gene expression [17]. For example, RA patients exhibit elevated protein biosynthesis activity, necessary for cell proliferation and production of proinflammatory cytokines such as interleukin (IL)-1β and tumor necrosis factor (TNF)-α [18]. These cytokines drive tissue destruction by activating matrix metalloproteinases, cathepsins, and promoting apoptosis [19]. Previous research shows that treatment with methotrexate or rituximab not only alters the expression of proinflammatory cytokine genes but also affects genes involved in core metabolic processes related to cell growth, proliferation, autophagy, apoptosis, and tissue degradation [20,21,22].

Cytokine biosynthesis in lymphocytes of patients with RA requires adequate energy in the form of adenosine triphosphate (ATP), primarily generated through glucose oxidation via glycolysis and the Krebs cycle [23]. Among glycolytic enzymes, PFKFB3 (6-phosphofructo-2-kinase/fructose-2,6-bisphosphatase 3) exhibits the highest kinase-phosphatase activity, enabling the maintenance of a high glycolytic rate [24]. Pyruvate produced by glycolysis is then decarboxylated to CO_2_ in the Krebs cycle, yielding reducing equivalents that are oxidized in the electron transport chain for ATP production [23]. In RA lymphocytes, glucose metabolism can also be diverted into the pentose phosphate pathway (PPP), which is activated by PFKFB3 [25]. The cell’s choice between the PPP and glycolysis depends on cytosolic nicotinamide adenine dinucleotide phosphate (NADP+) levels. When NADPH is depleted, glucose-6-phosphate dehydrogenase (G6PD), the key enzyme of the PPP, is activated [26]. NADPH plays a critical role as the main reducing agent protecting cells from oxidative stress and damage caused by free radicals formed during biosynthetic reactions [27]. Additionally, superoxide dismutase (SOD) functions as an important antioxidant enzyme neutralizing free radicals [28]. In RA lymphocytes, a disruption occurs in the intracellular localization of AMP-activated protein kinase (AMPK); it is no longer found on lysosomal membranes, leading to a loss of its regulatory functions. This mislocalization of AMPK can further impair cellular energy regulation and antioxidant defense in RA [17].

Lipid metabolism plays a key role in lymphocyte energy homeostasis and effector function [29]. Fatty acids (FAs) are utilized for both energy production via oxidation and the biosynthesis of cell membranes. During activation, effector lymphocytes upregulate FA synthesis while concurrently downregulating FA oxidation [30]. This synthesis is mediated by fatty acid synthase (FASN) [31], while the oxidation pathway requires the transport of FAs into the mitochondria by carnitine palmitoyltransferase (CPT), where they are converted into acetyl-CoA for use in the Krebs cycle [32].

Recently, autophagy has been suggested to play a role in the pathogenesis of rheumatoid arthritis (RA) [33]. Macroautophagy, a degradative process, allows cells to recruit and break down cytoplasmic components, providing nutrients and energy during starvation or clearing defective and toxic intracellular aggregates. Through autophagy recycling, macromolecules are degraded into glucose, free fatty acids, and amino acids, which then participate in various metabolic processes [34]. The key regulatory complex initiating autophagy is Unc-51-like kinase 1 (ULK1). ULK1 activity is inhibited by mTORC1 under nutrient-rich conditions but activated by AMPK during cellular stress or starvation [35]. Furthermore, synovial cell proliferation and bone homeostasis in RA are linked to the Wnt/β-catenin pathway, involving β-catenin, Wnt5A, and DKK1 activity [36,37]. Notably, evidence from animal studies indicates that β-catenin can directly bind to mTORC1, enhancing fatty acid synthesis [38].

We hypothesize that the systemic nature of RA contributes to the dysregulation of metabolic gene expression, potentially fueling inflammation, pain, and tissue destruction across multiple systems, including the nervous system. Because central sensitization (CS) arises from chronic inflammation, we propose that ongoing inflammatory activity may disrupt CNS metabolic homeostasis, with peripheral metabolic profiles potentially serving as biomarkers for this central dysregulation.

Consequently, this study aimed to investigate the relationship between CS severity and various clinical, immunological, and neuropsychiatric characteristics, alongside the expression of genes associated with inflammation, tissue destruction, and key carbohydrate and fatty acid metabolic pathways in PBMCs.

## 2. Results

### 2.1. Clinical, Immunological, and Neuropsychiatric Parameters of the Examined Patients with RA

All the examined patients with RA presented with moderate-to-high disease activity. Despite this, erythrocyte sedimentation rate (ESR) and C-reactive protein (CRP) levels were only marginally elevated. The mean disease duration was 114 months (range, 12–444 months), and body mass index (BMI) values were near-normal. The majority of patients tested positive for rheumatoid factor (RF) and anti-citrullinated protein antibodies (ACPA), presented with bone erosions, and were in an advanced or late stage of clinical disease. All patients reported morning stiffness, joint pain, and joint swelling, with moderate pain intensity recorded via the Visual Analogue Scale (VAS). Most patients were at radiographic stage II. While only 15.2% of patients were prescribed glucocorticoids, the majority received non-steroidal anti-inflammatory drugs (NSAIDs) (Table 1), specifically COX-1/2 inhibitors. Furthermore, all patients exhibited disturbances across various clinical assessments, including the Central Sensitization Inventory (CSI), Brief Pain Inventory (BPI), PainDETECT (neuropathic pain), Hospital Anxiety and Depression Scale (HADS), Fibromyalgia Impact (FIRST), fatigue scales (FSS and FACIT-F), Pain Catastrophizing Scale (PCS), and vitality assessments.

### 2.2. Clinical Characteristics of Patients with RA Related to CS Severity Score

Analysis of the clinical characteristics of patients with RA revealed no statistically significant differences across subgroups regarding age, disease duration, body mass index (BMI), disease activity (DAS28), CRP and ESR levels, pain severity (VAS and BPI), or joint counts (swollen or tender). Although the mean disease duration was 7.5–10 years and BMI values were slightly elevated, these differences were not statistically significant (Table 2).

It is important to note that RA disease activity was assessed using the DAS28 (Disease Activity Score-28), which integrates tender and swollen joint counts, patient global health assessments, and inflammation markers (ESR or CRP). Because these patients exhibited signs of central sensitization, their reported pain intensity was amplified, which artificially increased their DAS28 scores. Notably, these elevated DAS28 values occurred despite the absence of significantly high inflammatory markers, likely a result of treatment with NSAIDs and/or glucocorticoids. As shown in Table 2, patients consistently presented with moderate (3.2 < DAS28 < 5.1) to high (DAS28 > 5.1) disease activity, regardless of their central sensitization severity score, despite ongoing anti-inflammatory therapy. To eliminate treatment heterogeneity as a potential confounding factor, patients treated with disease-modifying antirheumatic drugs (DMARDs) were excluded from the study. The NSAIDs and glucocorticoids administered to the cohort effectively suppressed inflammation, as evidenced by the downregulated expression of proinflammatory cytokines and reduced CRP and ESR levels.

**Figure 1:**
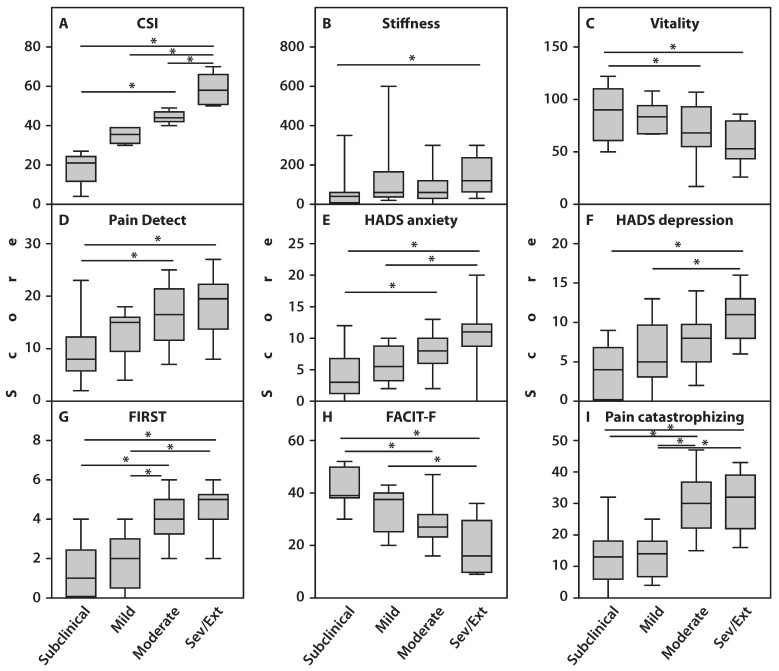
Clinical and neuropsychiatric parameters of patients with RA across different levels of CS severity. Parameters include (**A**) CSI score; (**B**) Stiffness; (**C**) Vitality; (**D**) PainDETECT; (**E**) HADS anxiety; (**F**) HADS depression; (**G**) FIRST; (**H**) FACIT-F; (**I**) Pain catastrophizing. An asterisk (*) indicates statistically significant differences (Kruskal–Wallis test) between the examined subgroups. Sev/Ext: patients with RA exhibiting severe and extreme CS scores.

However, vitality was significantly lower in patients with severe and extreme CS scores compared to those with subclinical (*p* = 0.01) or mild (*p* = 0.02) CS severity. Conversely, morning stiffness lasted significantly longer (*p* = 0.02) in patients with severe and extreme CS scores than in those with subclinical CS (Figure 1).

### 2.3. Neuropsychiatric Parameters in the Individual Subgroups of the Examined Patients with RA

SCI severity scores progressively increased across patient subsets. Specifically, CS severity score (*p* < 0.001), HADS-anxiety (*p* < 0.001), HADS-depression (*p* < 0.001), PainDetect (*p* = 0.003), FIRST (*p* < 0.001), and pain catastrophizing (*p* < 0.001) were significantly lower in patients with subclinical CS than in those with severe and extreme CS scores. Additionally, patients with subclinical CS showed significantly lower levels on the PainDetect (*p* = 0.03), HADS-anxiety (*p* = 0.04), FIRST (*p* < 0.001), and catastrophizing (*p* < 0.001) scales compared to patients with moderate CS. Patients with mild CS had lower scores in HADS-depression (*p* = 0.02), FIRST (*p* < 0.001), and pain catastrophizing (*p* < 0.001) compared to those with severe and extreme CS. Furthermore, patients with moderate CS scored significantly higher on FIRST (*p* < 0.001) and pain catastrophizing (*p* < 0.001) questionnaires than those with subclinical CS. No significant differences were found between subgroups regarding BPI severity and FSS scores. Fatigue scores, assessed by the FACIT-F questionnaire, showed a significant and gradual decrease (*p* < 0.01), indicating that fatigue severity increased with rising CS severity scores (Figure 1).

### 2.4. Changes in Gene Expression in PBMCs of Patients with RA Across Different Levels of CS Severity Score

**Figure 2:**
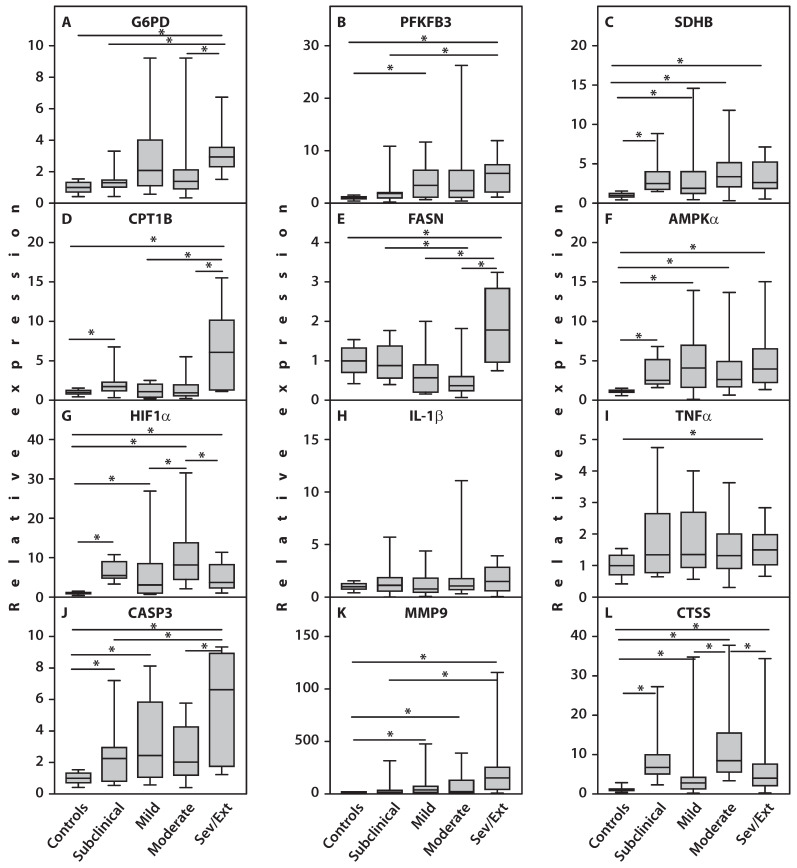
Expression of genes in PBMCs of patients with RA across different levels of CS severity. (**A**) G6PD; (**B**) PFKFB3; (**C**) SDHB; (**D**) CPT1B; (**E**) FASN; (**F**) AMPKα; (**G**) HIF1α; (**H**) IL-1β; (**I**) TNFα; (**J**) CASP3; (**K**) MMP9; (**L**) CTSS. An asterisk (*) indicates statistically significant differences (Kruskal–Wallis test) between the examined subsets. Sev/Ext: patients with RA exhibiting severe and extreme CS scores.

**Figure 3:**
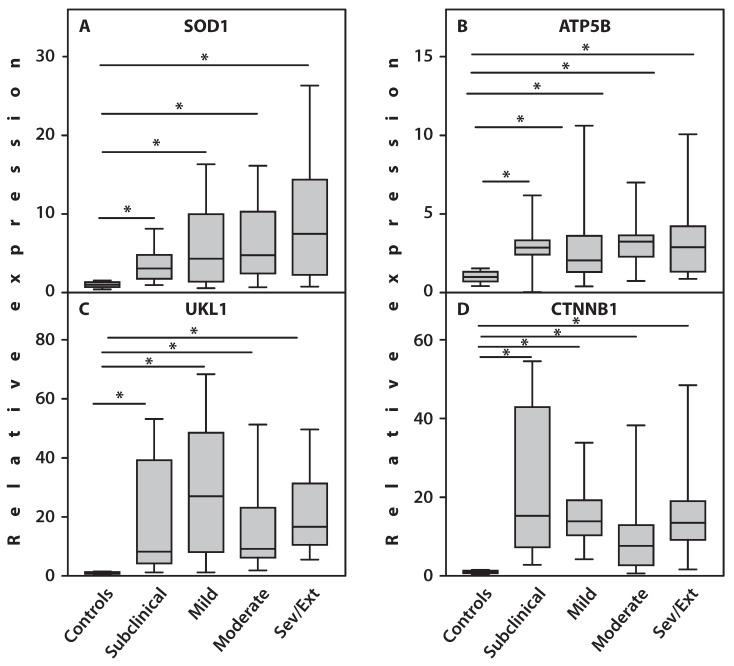
Expression of genes in PBMCs of patients with RA across different levels of CS severity. (**A**) SOD1; (**B**) ATP5B; (**C**) ULK1; (**D**) CTNNB1. An asterisk (*) indicates statistically significant differences (Kruskal–Wallis test) between the examined subsets. Sev/Ext: patients with RA exhibiting severe and extreme CS scores.

Importantly, gene expression of the proinflammatory cytokine IL-1β did not differ from healthy controls in any subgroup, and TNFα expression remained unchanged in three subgroups. Significant upregulation of TNFα gene expression was observed only in patients with severe and extreme CS scores (Figure 2). Analysis of gene expression across subgroups with varying CS scores revealed that patients with subclinical CS scores showed significantly increased expression of SOD1 (*p* = 0.001), AMPKα (*p* < 0.001), HIF1α (*p* = 0.001), SDHB (*p* = 0.001), ATP5B (*p* = 0.001), CPT1B (*p* = 0.01), CASP3 (*p* = 0.02), CTSS (*p* < 0.001), ULK1 (*p* < 0.001), and CTNNB1 (*p* < 0.001) compared to healthy controls. Since a CS score below 40 points is considered close to normal [39], these changes likely reflect RA pathology rather than scoring abnormalities (Figure 2 and Figure 3). In patients with mild and moderate CS scores, expression of the above genes—including MMP9—was also significantly increased (*p* = 0.001) versus controls. Additionally, CTSS (*p* = 0.002) and HIF1α (*p* = 0.03) expression were significantly higher in the moderate CS group compared to mild. In contrast, CPT1B expression in mild and moderate CS groups did not differ from controls.

The most significant changes in gene expression were observed in RA patients with severe and extreme CS severity scores. In these patients, expression of G6PD (*p* < 0.001), PFKFB3 (*p* = 0.03), CASP3 (*p* = 0.009), FASN (*p* = 0.005), and MMP9 (*p* = 0.007) was significantly higher compared to patients with subclinical CS scores. Additionally, CPT1B gene expression (*p* = 0.001) was significantly elevated in patients with severe and extreme CS compared to those with mild CS scores. Moreover, compared to the moderate CS group, RA patients with severe and extreme CS scores showed significantly increased expression of CASP3 (*p* = 0.02), CPT1B (*p* < 0.001), FASN (*p* < 0.001), and G6PD (*p* = 0.006), while the expression of HIF1α (*p* = 0.03) and CTSS (*p* = 0.03) was significantly decreased (Figure 2).

In summary, the highest statistical significance was observed for increased expression of G6PD (*p* < 0.001), CASP3 (*p* = 0.009), MMP9 (*p* = 0.007), CPT1B (*p* < 0.001), and FASN (*p* < 0.001) in the severe/extreme CS subgroup compared to patients with lower severity scores.

### 2.5. Protein Levels of the Examined Genes in Isolated PBMCs

**Figure 4:**
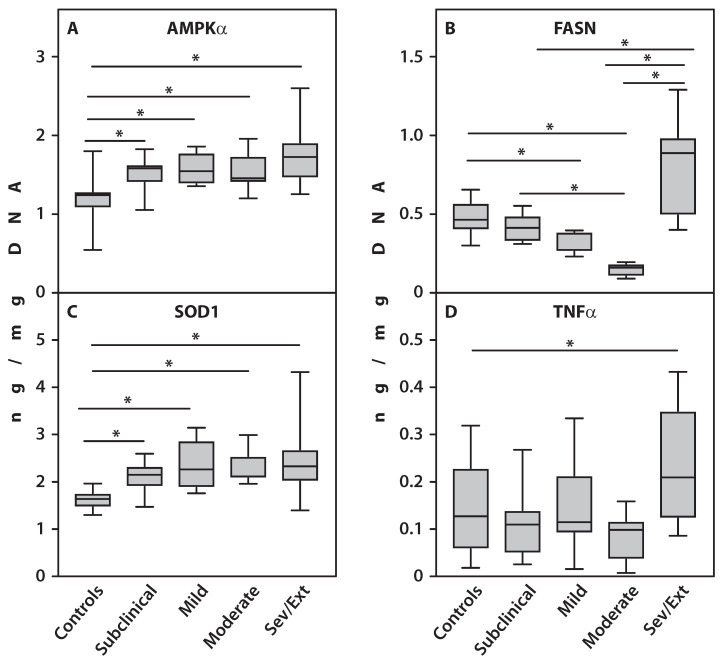
Protein concentrations of AMPKα (**A**), FASN (**B**), SOD1 (**C**), and TNFα (**D**) measured by ELISA in PBMCs from patients with RA across varying degrees of CS severity. An asterisk (*) indicates statistically significant differences (Kruskal–Wallis test) between the examined subsets. Sev/Ext: patients with RA exhibiting severe and extreme CS scores.

To assess the clinical significance of the relative expression of the examined genes in the PBMCs of RA patients, we measured protein concentrations of AMPKα, FASN, SOD1, and TNFα in the PBMC fraction. Protein levels of AMPKα and SOD1 were significantly higher (*p* < 0.001) in all patient subgroups compared to healthy controls (Figure 4). TNFα protein expression was significantly increased (*p* = 0.002) only in patients with severe and extreme CS scores compared to controls. FASN protein concentrations were significantly lower in patients with moderate (*p* < 0.001) and subclinical (*p* = 0.03) CS scores compared to controls. Furthermore, patients with moderate CS scores showed lower FASN levels than those with subclinical scores (*p* = 0.01). Conversely, patients with severe and extreme CS scores exhibited significantly higher FASN protein levels (*p* < 0.001) compared to subclinical (*p* = 0.01), mild (*p* < 0.001), and moderate (*p* < 0.001) subgroups (Figure 4).

In Figure 4, we demonstrate that the gene expression results align closely with the protein expression data obtained from ELISA analysis of PBMC samples from the same patients. These analyses revealed significant differences in the gene expression levels of AMPKα, FASN, SOD1, and TNFα among the RA patient subgroups, which were confirmed by corresponding significant variations in protein concentrations within those subgroups.

### 2.6. Correlation Analyses of Clinical Parameters in RA Patients with Neuropsychiatric Characteristics

Correlation analyses of clinical parameters in RA patients with neuropsychiatric characteristics revealed a moderate positive correlation between the severity of CS signs and most examined parameters. Notably, a very strong correlation was found with the level of fibromyalgia. The only negative correlation with CS severity was observed with the FACIT-F score. Additionally, most neuropsychiatric parameters showed moderate to weak positive correlations with morning stiffness. Other clinical correlations included a positive association between ESR levels and HADS depression scores, as well as between CRP levels and pain severity measured by the BPI questionnaire. Pain severity measured by VAS negatively correlated with the FACIT-F score (Table 3).

### 2.7. Correlation Analyses of Gene Expression with Clinical and Neuropsychiatric Characteristics of Patients with RA (n = 59)

Correlation coefficient analyses revealed moderate to weak positive correlations between the expression of most examined genes and clinical parameters. Notably, only ATP5B expression showed a negative correlation with CRP levels. The expression of G6PD, UCP2, and CASP3 genes was associated with morning stiffness, while MMP-9, PFKFB3, SDHB, and AMPKα expression correlated with pain measured by VAS. Additionally, MMP-9 and PFKFB3 expressions correlated with most clinical parameters, and ULK1 expression positively correlated with the number of swollen joints and DAS28 (CRP) (Table 4).

Furthermore, moderate and weak positive correlations were observed between the expression of G6PD, CASP3, MMP-9, PFKFB3, HIF1α, CPT1B, and ULK1 genes and the neuropsychiatric features of patients with RA. A negative correlation was found between HIF1α expression and HADS depression scores, as well as between CASP3, MMP-9, CPT1B gene expressions and the FACIT-F score (Table 5).

### 2.8. Protein–Protein Interaction (PPI) Network Construction

**Figure 5:**
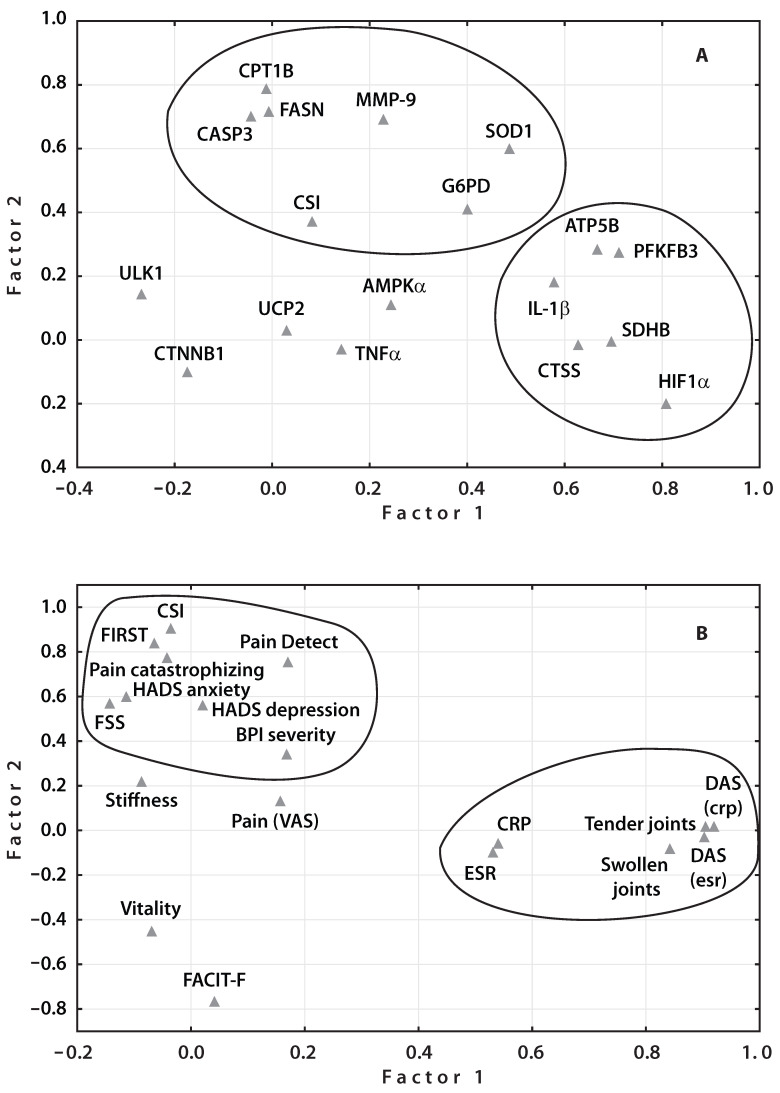
Principal component analysis (PCA): (**A**) Gene expression subgroups; factor analysis identified a primary factor (Factor 2) containing six genes in patients with RA and CSI scores > 40, and a second primary factor (Factor 1) containing six genes in patients with RA and CSI scores < 40. (**B**) Clinical and neuropsychiatric characteristic subgroups; factor analysis identified a primary factor (Factor 1) comprising neuropsychiatric traits in patients with RA and CSI scores > 40, and a second factor (Factor 2) comprising clinical characteristics in patients with RA and CSI scores < 40.

Factor analysis, specifically principal component analysis (PCA), was used to identify genes whose expression is primarily associated with central sensitization (CS) among the examined genes. Since CS is considered pathological starting from a moderate CSI score of 40 points or higher, patients were divided into two subgroups: CS-positive (*n* = 25) with CSI scores above 40 (linked to Factor 2), and CS-negative (*n* = 34) with scores below 40 (linked to Factor 1). Genes with factor loadings above 0.4 that showed the strongest association with CS included CPT1B, FASN, CASP3, MMP-9, G6PD, and SOD1. In contrast, ATP5B, PFKFB3, SDHB, CTSS, HIF1α, and IL-1β expressions were associated with the CS-negative subgroup (Figure 5A).

**Figure 6:**
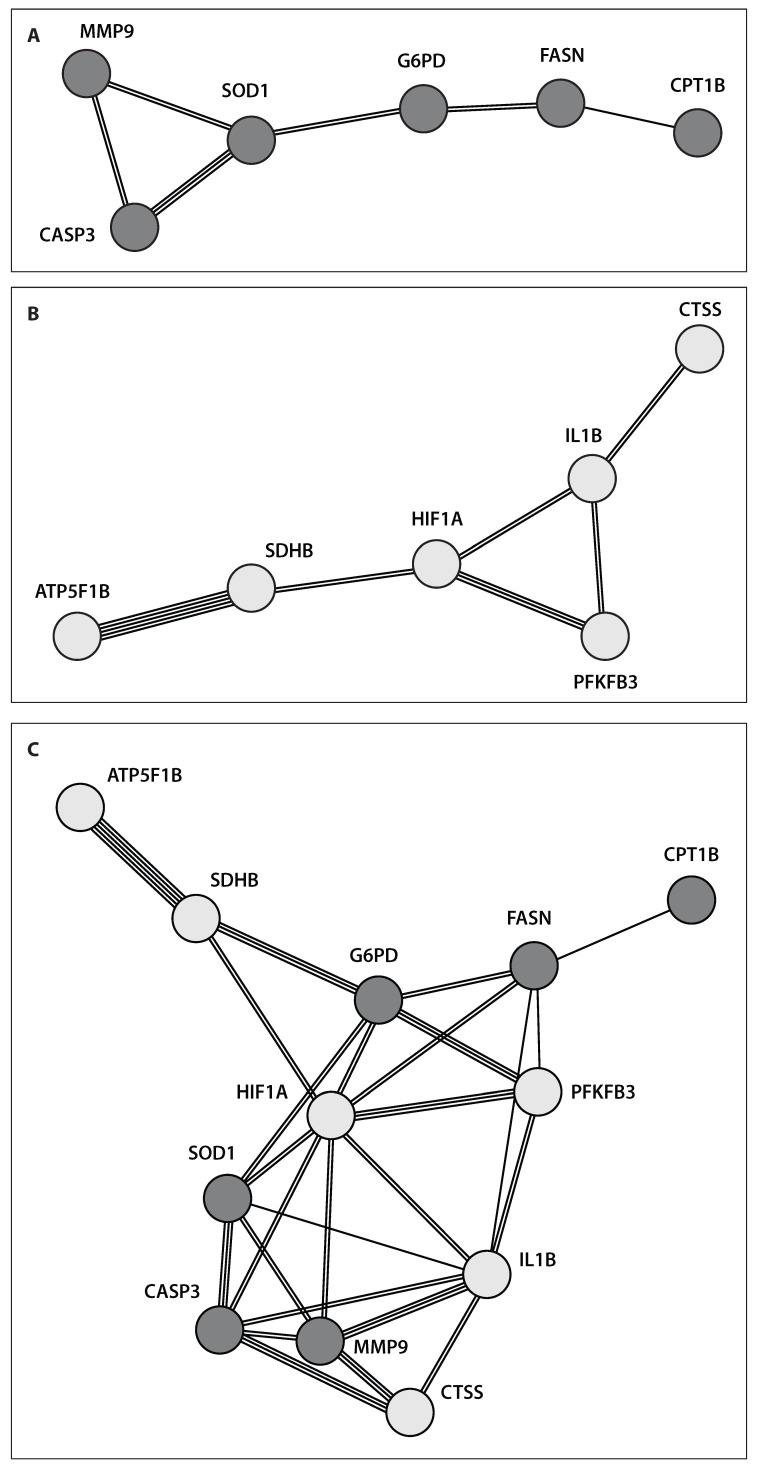
Protein–protein interaction (PPI) networks for gene expressions in the peripheral blood of patients with RA stratified by Central Sensitization (CS) severity score: (**A**) CS severity score > 40 (grey circles), (**B**) CS severity score < 40 (white circles), and (**C**) the integrated network of all identified genes.

Principal component analysis (PCA) of clinical and neuropsychiatric characteristics in RA patients, divided into CS-positive and CS-negative groups, showed that neuropsychiatric measures—assessed by FIRST, HADS anxiety, HADS depression, Pain DETECT, FSS, Pain Catastrophizing, and BPI severity questionnaires—had the strongest association with CS, with factor loadings above 0.3. Conversely, clinical RA traits such as DAS (ESR), DAS (CRP), ESR, CRP, tender joints, and swollen joints were primarily associated with Factor 1 (factor loadings above 0.4) in the CS-negative subgroup (Figure 5B). The PPI network analysis using the STRING database aimed to map the interactions among the identified proteins. PPIs represent specific physical contacts between proteins, mediated by biochemical forces like electrostatic interactions, hydrogen bonds, and hydrophobic effects. Node connectivity in the network corresponds to how many interactions a protein has. In this study, PPI analysis of the 12 identified genes revealed a tightly interconnected network. Notably, in RA patients with CS (CSI > 40), there was a strong interaction between CASP3 and MMP9 (interaction score 0.806), genes linked to apoptosis and extracellular matrix degradation, respectively (Figure 6A). These genes also connected to pathways involving fatty acid metabolism (FASN, CPT1B), the pentose phosphate pathway (G6PD), and antioxidant defense (SOD1). However, in the broader patient group, these interaction scores ranged from 0.413 to 0.624, indicating weaker connectivity compared to patients with CS manifestations.

In contrast, patients with RA and CS score below 40 exhibited the strongest association between the ATP5B and SDHB genes (interaction score [IS] = 0.879), along with notable connections between SDHB and HIF1α (IS = 0.686), and between HIF1α and PFKFB3 (IS = 0.781). These genes are key players in energy production through the Krebs cycle and glycolysis. Additionally, these interactions were linked to gene expressions related to inflammation (IL1B) and pain (CTSS). Interaction scores among these genes ranged from 0.668 to 0.781 (Figure 6B). The integrated network of all 12 genes revealed that signaling pathways associated with CS manifestations and those of the non-CS (healthy) phenotype did not overlap significantly, highlighting distinct molecular disruptions tied to CS traits (Figure 6C). Notably, the highest factor 1 loading (0.858) was observed for HIF1α gene expression, underscoring its role as a key regulator connected primarily with G6PD and SOD1 genes. This suggests that CS is likely linked to a hypoxic environment accompanied by oxidative stress and activation of the pentose phosphate pathway (PPP).

## 3. Discussion

In recent years, it has become increasingly evident that the immune and central nervous systems interact closely [40]. As a result, patients with chronic inflammatory diseases like rheumatoid arthritis (RA) often experience neuropsychiatric symptoms such as depression, anxiety, and fatigue. Gene expression analysis in RA patients with a subclinical CS score showed that antirheumatic therapy was generally effective, as the expression of anti-inflammatory cytokines (TNFα, IL-1β) was comparable to that of healthy controls. This aligns with previous findings reporting similar TNFα levels in patients with fibromyalgia and healthy individuals [41]. Notably, our earlier studies linked successful antirheumatic treatment to decreased expression of proinflammatory cytokine genes, accompanied by reduced inflammation, pain, and tissue damage [20,21,22]. However, in patients with subclinical CS scores, low expression of these genes did not correlate with symptom relief or reduced disease activity, suggesting other mechanisms may hinder therapeutic efficacy. Additionally, tissue destruction was likely suppressed since metalloproteinase MMP-9 expression in these patients did not differ from controls.

Since we previously noted that antirheumatic therapy alters the expression of genes involved in central metabolic processes [20,21,22], the observed discrepancy may result from both the activation of alternative signaling pathways and changes in genes related to core metabolism. Specifically, the expression levels of genes linked to glycolysis (PFKFB3), the pentose phosphate pathway (G6PD), and fatty acid synthesis (FASN) were similar in patients with a subclinical CS score and healthy controls. Despite this, immune cells from these patients with RA showed signs of energy deprivation, as indicated by increased AMPKα expression [42]. This occurred even though electron transport chain (ETC) activity was elevated, evidenced by higher expression of ATP5B and the uncoupling protein UCP2, which reduces free radical production, along with superoxide dismutase SOD1 to neutralize reactive oxygen species [43]. These findings suggest a hypoxic environment, supported by upregulated hypoxia-inducible factor HIF1α [44]. HIF1α can regulate the Krebs cycle’s descending branch by promoting succinate production through the reverse succinate dehydrogenase reaction, as shown by increased SDHB expression [45]. Therefore, the ATP deficiency observed may stem from Krebs cycle disruption despite active ETC function.

Energy deficiency may be linked to increased apoptosis, as shown by elevated CASP3 gene expression in patients with a subclinical CS score compared to controls. This is accompanied by higher expression of CPT1B, a gene involved in fatty acid oxidation [46], which helps compensate for the shortage of organic acids needed for synthesizing new cellular structures [18]. Additionally, significantly increased expression of CTSS, a marker associated with neuropathic pain [47], in these patients suggests a connection to CS characteristics.

Patients with RA and mild to moderate CS scores also showed elevated expression of these genes, while proinflammatory cytokine levels remained similar to controls. Notably, MMP-9 expression was significantly higher in these patients, which is important because MMP-9 not only degrades the extracellular matrix but is also linked to hypernociception [48]. This may develop gradually alongside worsening neuropsychiatric symptoms associated with CS, supported by the finding that CTSS expression is significantly greater in moderate versus mild CS cases. Furthermore, patients with RA and a mild CS score exhibited a significant increase in PFKFB3 gene expression, which activates the pentose phosphate pathway (PPP). This suggests a heightened demand for NADPH, required for anabolic processes, free radical neutralization during oxidative stress, and as energy for synthesizing fatty acids, nucleotides, and other cellular components [27].

It is important to note that the increased expression of these genes correlates with higher scores on measures of neuropathic pain (Pain DETECT), fibromyalgia (FIRST), and pain catastrophizing. Additionally, patients with moderate CS severity showed a significant increase in fatigue severity (FSS), accompanied by a decrease in the “Fatigue in Chronic Diseases” index (FACIT-F), indicating overall weakness. Since CS impairment is considered pathological starting at a moderate CS score (40 points or more) [39], it is significant that this subgroup exhibits notably higher expression of the HIF1α and CTSS genes compared to those with mild CS scores. This points to increased hypoxia and a greater dependence on glycolysis for energy production. Furthermore, the marked rise in CTSS expression in moderate CS patients suggests an amplified role for neuropathic mechanisms [47].

The most significant gene expression changes in RA patients with severe and extreme CS severity scores are linked to increased proinflammatory activity, demonstrated by elevated TNFα gene expression compared to healthy controls. There is also enhanced metabolic activity, shown by upregulation of genes involved in the pentose phosphate pathway (G6PD) and fatty acid metabolism, with increased expression of CPT1B and FASN [31,32] compared to patients with moderate CS scores. Genes related to tissue destruction and apoptosis, such as MMP-9 and CASP3, were also upregulated. Markers of neuropathic pain, indicated by higher MMP-9 and CTSS expression [48], correlate with a notably greater perception of stiffness in patients with severe or extreme CS versus moderate CS. These results suggest a strong connection between central metabolic disturbances and neuropsychiatric factors in patients with high CS severity. This is further confirmed by increased anxiety and depression (HADS scores) and reduced fatigue (FACIT-F scores) in this subgroup compared to those with moderate scores.

PCA analyses integrating gene expression with clinical and neuropsychiatric data identified that the primary factor associated with CS involved both neuropsychiatric symptoms and expression of CPT1B, FASN, CASP3, MMP-9, G6PD, and SOD1. Thus, alterations in fatty acid metabolism, apoptosis, neuropathic pain mechanisms, the pentose phosphate pathway, and antioxidant defense are closely linked to central sensitization manifestations in patients with RA.

Identification of HIF1α as a central regulatory node linked to both CS-related and RA-related gene expression is expected, given its established role in hypoxia-driven pathological neurodegeneration and inflammation in RA, including synovial, bone, and cartilage damage [49]. Our study’s findings further support this association, as HIF1α expression correlates with key CS-related genes. For instance, in cultured macrophages, upregulation of HIF1α modifies glucose metabolism by inducing G6PD expression [50]. HIF1α also regulates lipid metabolism, shown by its ability to increase FASN expression while repressing CPT1, demonstrated in non-small cell lung cancer models [51]. Additionally, impaired antioxidant defense marked by SOD1 inhibition aligns with elevated HIF1α expression in autoimmune diseases [52]. HIF1α’s role in controlling apoptosis and caspase 3 expression has been documented in cancer research [49,53]. In RA pathogenesis, hypoxia contributes to the tumor-like behavior of fibroblast-like synoviocytes, accompanied by upregulated MMP-9 expression [54]. However, these processes have primarily been linked to localized hypoxic conditions within affected tissues [45].

Indeed, since this study is cross-sectional, we cannot establish causation regarding the metabolic findings and therefore can only speculate. All patients examined belong to a difficult-to-treat cohort, with disease durations of 90–120 months. They exhibited various degrees of central sensitization (CS) disturbances, ranging from subclinical to extreme severity, but CS pathology is defined only at a CS score greater than 40. As a systemic disease, rheumatoid arthritis affects every cell, tissue, and organ, including the nervous system, making metabolic reprogramming possible across the entire body. Variations in RA severity and inflammation in different tissues—including the nervous system—may result from genetic, environmental, and social factors. Since the examined subgroups showed no significant differences in disease duration or other RA clinical manifestations, CS development could reflect individual differences in nervous system adaptability. Moreover, the upregulation of CS-associated genes across all subgroups compared to healthy controls suggests that CNS-driven dysregulation may influence peripheral metabolic profiles even at subclinical and mild CS levels. Patients with CS scores above 40 differ primarily by a quantitative increase in the expression of these genes rather than by qualitative differences.

There are some limitations to our study. Firstly, the sample size is small due to the specific selection of patients who had never been treated with biological (b)DMARDs or JAK inhibitors, and who also avoided conventional synthetic (cs)DMARDs for the last three months to reduce patient heterogeneity. All patients were treated with NSAIDs, which likely have similar effects as they are COX-1/2 inhibitors. Additionally, the range of disease duration and slightly elevated BMI values did not differ significantly between subgroups. However, variability in these traits as well as smoking, age, gender or comorbidities could still influence gene expression results and act as confounding factors. Secondly, as an observational cross-sectional study, it does not allow us to establish a causal relationship between metabolic alterations and CS-related stress responses. This study only identifies associations between CS disturbances and changes in gene expression related to fatty acid metabolism, oxidative stress, and ECM degradation/neuropathic pain. To address these limitations, a well-designed longitudinal study with a larger population will be necessary.

## 4. Materials and Methods

### 4.1. Patients

The study included 59 patients with RA, among whom there were 11 men and 48 women, with a mean age of 52.0 ± 13.0 years (range 19–75 years). The patients were treated at the Nasonova Research Institute of Rheumatology between 2021 and 2023. Study Protocol No. 12, dated 23 December 2021, was approved by the local ethics committee at the Nasonova Research Institute of Rheumatology, and written informed consent was obtained from all participants.

Inclusion criteria for the study were as follows:

Age ≥ 18 years

A confirmed diagnosis of RA according to the 2010 ACR/EULAR criteria

Moderate to high disease activity (DAS28-CRP ≥ 3.2)

Insufficient response to current therapy

Informed consent obtained from the patient

Exclusion criteria were:

Pregnancy or lactation

Severe functional impairment or comorbidities that prevent regular monitoring or the prescription of antirheumatic drugs

No prescription of biologic agents or genetically engineered biological drugs. The patients treated with csDMARDs or bDMARDs or JAK inhibitors in the last 3 months were excluded. However, previously csDMARDs (Methotrexate (primarily), Leflunomide, Hydroxychloroquine, or Sulfasalazine) were prescribed to the examined patients in the course of RA disease.

All RA patients were divided into four subgroups based on their CS severity score according to the Central Sensitization Inventory (CSI) Part 1 [39]:

Subclinical (0–29 points), *n* = 13

Mild (30–39 points), *n* = 12

Moderate (40–49 points), *n* = 20

Severe (50–59 points), *n* = 9

Extreme (60–100 points), *n* = 5

The control group consisted of 26 individuals (7 men and 19 women) with a mean age of 53.8 ± 12.0 years (range 19–69 years), who did not have chronic pain, acute infections, or a family history of autoimmune diseases.

### 4.2. Demographic, Clinical, and Immunological Assessment of Patients with RA

The following parameters were recorded: age, gender, body mass index (BMI), disease duration, Steinbrocker radiographic stage [55], duration of morning stiffness (minutes), and disease activity scores (DAS-CRP and DAS-ESR) using a modified 28-joint index [56]. Serum C-reactive protein (CRP) levels (cutoff value: 5 mg/L) and IgM rheumatoid factor (RF) concentrations (standard cutoff value: 15 mU/mL) were measured by nephelometry using a BN-100 analyzer (Dade Behring, Ashbourne, Germany). Anti-citrullinated protein autoantibodies (ACPA) were determined using ELISA according to the manufacturer’s instructions, with a cutoff value of 5 U/mL for a positive antibody result (Axis Shield Diagnostics Limited, Dundee, UK).

### 4.3. Assessment of the Severity of CS Symptoms, Pain, Anxiety, Depression, Fatigue, and Fibromyalgia

The Central Sensitization Inventory (CSI, Part 1) scale was used to assess symptoms of central sensitization (CS). Pain intensity was evaluated with the Brief Pain Inventory (BPI) questionnaire. Neuropathic pain symptoms were identified using the Pain DETECT questionnaire. Anxiety and depression levels were measured with the Hospital Anxiety and Depression Scale (HADS). Fatigue severity was assessed using the Fatigue Severity Scale (FSS), while fibromyalgia symptoms were screened with the Fibromyalgia Rapid Screening Tool (FIRST). To evaluate fatigue specifically in patients with chronic illnesses, the Functional Assessment of Chronic Illness Therapy-Fatigue (FACIT-F) questionnaire was employed. Notably, lower FACIT-F scores indicate greater symptom severity, whereas higher scores on all the other questionnaires reflect increased symptom severity.

### 4.4. Quantification of Protein Levels by the Enzyme-Linked Immunosorbent Assay (ELISA)

We collected 10 mL of peripheral blood in vacutainers containing ethylenediaminetetraacetic acid (EDTA) (Sigma-Aldrich, Inc., St. Louis, MO, USA) to prevent coagulation and preserve sample integrity for subsequent analyses. Blood was drawn in a standard manner between 07:00 and 09:00 AM after an overnight fast and before breakfast. Whole blood was separated using a Ficoll density gradient. Peripheral blood mononuclear cells (PBMCs), which have a lower density than Ficoll (1.077 g/mL), were isolated by centrifugation after layering diluted blood over the Ficoll. Following centrifugation, layers formed consisting of thrombocyte-enriched plasma, PBMCs at the interphase, and a pellet containing granulocytes above the red blood cells. PBMCs from the interphase were carefully isolated and washed twice with phosphate-buffered saline (PBS). Erythrocytes were lysed using a hypotonic buffer (1.6 mM EDTA, 10 mM KHCO_3_, 153 mM NH_4_Cl, pH 7.4) at a 3:1 volume ratio. The isolated PBMCs were then frozen and stored at −80 °C until protein extraction.

Concentrations of TNFα (HEA133Hu), SOD1 (SES134Hu), AMPKα (SEA679Hu), and FASN (SEC470Hu) in the isolated PBMCs were measured using commercial ELISA kits (Cloud-Clone Corp., Wuhan, China) following the manufacturer’s protocols. Results were normalized per μg of DNA measured in PBMC lysates. Lysates were prepared with a Cell Extraction Buffer containing multiple components (including 10 mM Tris pH 7.4, 100 mM NaCl, 1 mM EDTA, 1% Triton X-100, 10% glycerol, 0.1% SDS, and 0.5% deoxycholate (Invitrogen, Camarillo, CA, USA) supplemented with Protease Inhibitor Cocktail (Sigma-Aldrich, Inc., St. Louis, MO, USA) and 1 mM PMSF (Sigma-Aldrich, Inc., St. Louis, MO, USA). Total DNA content in PBMC lysates was quantified spectrophotometrically using a GeneQuant device (Amersham Biosciences, Cambridge, UK), ensuring accuracy in expressing results per μg of DNA.

### 4.5. Total RNA Isolation, Reverse Transcriptase (RT) Reaction, and Quantitative Real-Time Polymerase Chain Reaction (PCR)

Total RNA was isolated from each freshly obtained blood sample, and reverse transcription (RT) was performed. The resulting cDNA was stored at −20 °C as previously described [57]. Pre-designed primer and probe sets were used for analysis with the TaqMan assay (Applied Biosystems, Foster City, CA, USA): PFKFB3(Hs00998698_m1); G6PD (Hs00166169_m1); ATP5B (Hs00969569_m1); SDHB (Hs01042482_m1); UCP2 (Hs01075227_m1); AMPKα (Hs01562315_m1); HIF1α (Hs00936368_m1); SOD1 (Hs00533490_m1); FASN (Hs01005622_m1); CPT1B (Hs00189258_m1); CTSS (Hs00175407_m1); CASP3 (Hs00263337 m1), TNF*α* (Hs00174128 m1), IL-1*β* (Hs00174097 m1), MMP9 (Hs00234579 m1), Ulk1 (Hs00177504), CTNNB1 (Hs00355045_m1). *β*-Actin served as an endogenous control.

mRNA quantification was carried out using a QuantStudio 5 instrument (Applied Biosystems, Foster City, CA, USA), following the protocol detailed in Ref. [57]. Relative mRNA expression was calculated using the delta-delta CT method according to the manufacturer’s guidelines. The delta CT value was obtained by subtracting the CT value of the housekeeping gene β-Actin from the CT value of each sample. Then, the delta-delta CT value was determined by subtracting the delta CT of the control (healthy individuals) from that of each OA patient. All PCRs were conducted in duplicate to ensure reliability.

### 4.6. Statistical Analysis

The normality of data distribution was assessed using the Kolmogorov–Smirnov test. Data are presented as medians [25th, 75th percentile]. The Mann–Whitney U test was used for comparisons between two groups, while the nonparametric Kruskal–Wallis test with Bonferroni correction was employed for comparisons among more than two groups. The protein–protein interaction (PPI) network was constructed using the Search Tool for the Retrieval of Interacting Genes (STRING, version 12.0). Statistical analyses were performed using Statistica for Windows (StatSoft Inc., version 10, Tulsa, OK, USA) and SPSS version 19 (IBM, Armonk, NY, USA). All experiments were performed in triplicate. A *p*-value < 0.05 was considered statistically significant. Statistically significant differences compared to the control group are indicated by an asterisk (*).

## 5. Conclusions

Our study demonstrates that increased central sensitization (CS) in RA patients is characterized by the low gene expression of proinflammatory cytokines IL-1β and TNFα. At the same time, genes linked to tissue destruction, hypernociception, and cell death (MMP9 and CASP3) were activated. We also observed an upregulation of FASN, suggesting active production of new cellular components. The ATP required for these processes may be supplied by the upregulation of CPT1B (fatty acid breakdown) and the activation of glycolysis and the pentose phosphate pathway via PFKFB3. To counter the potential for excessive free radical production caused by this metabolic surge, there was a compensatory increase in antioxidant genes G6PD and SOD1. This gene expression dysregulation was most evident in patients with severe and extreme CS, who also showed the greatest neuropsychiatric impairment. Consequently, CS-related disturbances appear to worsen disease severity in RA patients, independent of standard antirheumatic therapy. These insights into the relationship between central sensitization and metabolic gene expression could guide the development of new treatments that address both pain and metabolic dysfunction.

## Figures and Tables

**Table 1:** Clinical immunological, and neuropsychiatric parameters of the examined patients with RA.

	Normal Values	Median [IQR] (% Patients)
Disease duration, months		114 [60; 177]
BMI, kg/m^2^	<24.9	26.3 [21.3; 30.1]
IgM RF, MU/mL	<14	46 (77.9%)
ACPA, U/mL	<20	42 (71.1%)
CRP, mg/L	<5	6.7 [3.1; 16.3]
ESR, mm/h	<15–20	18 [13; 35]
DAS28 (ESR)	<2.6	4.82 [4.19; 5.76]
DAS28 (CRP)	<2.6	4.59 [4.02; 5.46]
Stiffness, min	0	60 [30; 120]
Number of swollen joints	0	4 [3; 6]
Number of tender joints	0	7 [5; 11]
Pain (VAS), mm	0	60 [50; 70]
Erosions	0	47 (81%)
Clinical stage:		
Early		1 (1.6%)
Advanced		40 (67.8%)
Late		18 (30.1%)
Radiographic stage:		
I		3 (5%)
II		31 (52.5%)
III		8 (13.5%)
IV		16 (27.1%)
**Scores:**		
CSI	<40	42 [30; 49]
Pain DETECT	<12	14 [10; 20]
HADS anxiety	<7	8 [4; 1]
HADS depression	<7	8 [4; 11]
FSS	0	43 [30; 52]
FIRST	<4	4 [2; 5]
FACIT-F	>4	30 [23; 38.25]
Pain catastrophizing	<30	22.5 [14; 32.25]
Vitality	80	72 [55.75; 91.50]
BPI severity	0	4.5 [2.75; 5.5]
**Therapy:**		
Glucocorticoids in the last 6 months		9 (15.2%)
NSAIDS in the last 3 months:		
Nimesulide		16 (27.1%)
Aceclofenac		1 (1.7%)
Ibuprophen		6 (10.1%)
Diclofenac		8 (13.6%)
Meloxicam		13 (22.1%)
Naproxen		1 (1.7%)
Etoricoxib		1 (1.7%)
Ketorolac		1 (1.7%)
Ketoprofen		1 (1.7%)
Celecoxib		2 (3.4%)

**Table 2:** Clinical traits of patients with RA in relation to CS severity score.

CS Score	Subclinical Median [IQR]	Mild Median [IQR]	Moderate Median [IQR]	Severe/Extreme Median [IQR]
Pain (VAS)	60 [60; 60]	60 [50; 60]	60 [50; 60]	60 [51; 68.7]
CRP	6.3 [3.2; 22.8]	4.55 [3.87; 13.5]	7.3 [2.87; 17.7]	7.08 [2.96; 12.9]
ESR	14.0 [13.0; 35.0]	17.0 [7.0; 35.5]	18.5 [10.7; 42.0]	17.0 [13.0; 26.8]
DAS (CRP)	4.49 [4.1; 4.99] Nmod = 11 Nhigh = 2	4.74 [4.27; 5.37] Nmod = 7 Nhigh = 5	4.56 [4.11; 6.32] Nmod = 12 Nhigh = 8	4.7 [4.0; 5.45] Nmod = 11 Nhigh = 4
DAS (ESR)	4.65 [4.4; 5.51] Nmod = 8 Nhigh = 5	4.86 [4.1; 5.32] Nmod = 6 Nhigh = 6	4.65 [4.0; 6.02] Nmod = 12 Nhigh = 8	4.82 [7.2; 5.65] Nmod = 9 Nhigh = 6
Number of swollen joints	4.0 [3.0; 5.0]	4.5 [2.75; 9.25]	4.0 [3.0; 6.5]	4.5 [2.62; 6.0]
Number of tender joints	7.0 [5.0; 11.0]	6.5 [4.0; 8.75]	7.0 [5.0; 11.75]	6.75 [5.1; 9.62]
BPI severity	2.75 [1.5; 5.25]	4.12 [2.0; 5.0]	5.0 [3.68; 5.62]	4.62 [3.25; 5.27]
FSS	3.33 [1.67; 5.33]	4.33 [3.22; 4.78]	5.05 [4.27; 5.89]	5.52 [4.46; 6.24]
Disease duration, months	120 [64; 174]	90 [42; 165]	120 [24; 128]	120 [54; 240]
BMI	25.6 [21.2; 29.9]	24.9 [21.2; 27.6]	26.7 [22.0; 32.4]	25.6 [19.0; 29.3]

Nmod, number of patients with moderate disease activity; Nhigh, number of patients with high disease activity.

**Table 3:** Correlation coefficients (Spearman) and their significance (*p*) for clinical and neuropsychiatric parameters in patients with RA (*n* = 59).

	SCI	Pain DETECT	HADS Anxiety	HADS Depression	FSS	FIRST	FACIT-F	Pain Catastro-Phizing	BPI Severity
CSI		0.507 *p* < 0.001	0.619 *p* < 0.001	0.589 *p* < 0.001	0.402 *p* = 0.002	0.723 *p* < 0.001	−0.658 *p* < 0.001	0.654 *p* < 0.001	0.420 *p* < 0.002
Stiffness	0.305 *p* = 0.019			0.390 *p* = 0.002		0.299 *p* = 0.021	−0.432 *p* = 0.001	0.374 *p* = 0.003	0.300 *p* = 0.02
Pain (VAS)							−0.269 *p* = 0.04		
ESR				0.261 *p* = 0.046					
CRP									0.304 *p* = 0.01

**Table 4:** Correlation coefficients (Spearman) and their significance (*p*) for gene expression with clinical characteristics in patients with RA (*n* = 59).

Gene	Stiffness	Number of Swollen Joints	Number of Tender Joints	Pain (VAS)	ESR	CRP	DAS28 (ESR)	DAS28 (CRP)
G6PD	0.397 *p* = 0.002							
UCP2	0.278 *p* = 0.035							
CASP3	0.285 *p* = 0.02							
MMP-9		0.272 *p* = 0.037	0.306 *p* = 0.002	0.259 *p* = 0.048	0.354 *p* = 0.006	0.306 *p* = 0.019	0.364 *p* = 0.004	0.371 *p* = 0.004
PFKFB3			0.291 *p* = 0.025	0.261 *p* = 0.045		0.308 *p* = 0.018	0.335 *p* = 0.01	0.330 *p* = 0.011
HIF1α			0.330 *p* = 0.008					
ATP5B						−0.286 *p* = 0.028		
IL-1β							0.269 *p* = 0.04	
AMPKα				0.280 *p* = 0.032				
SDHB				0.259 *p* = 0.048				
ULK1		0.420 *p* < 0.001						0.308 *p* = 0.01

**Table 5:** Correlation coefficients (Spearman’s rank order) and their significance (*p*) for gene expression with neuropsychiatric characteristics in patients with RA (*n* = 59).

Gene	CSI	HADS Anxiety	HADS Depression	FSS	FIRST	FACIT-F	BPI Severity	Pain DETECT
G6PD	0.339 *p* = 0.009		0.327 *p* = 0.01		0.282 *p* = 0.03			
CASP3	0.380 *p* = 0.003	0.272 *p* = 0.039	0.448 *p* < 0.001		0.277 *p* = 0.036	−0.334 *p* = 0.014		
MMP-9	0.375 *p* = 0.003		0.352 *p* = 0.006			−0.281 *p* = 0.04		
PFKFB3	0.322 *p* = 0.013						0.304 *p* = 0.01	
CPT1B	0.308 *p* = 0.017			0.336 *p* = 0.009		−0.276 *p* = 0.043		
HIF1α			−0.282 *p* = 0.031	0.328 *p* = 0.011				
ULK1								0.284 *p* = 0.02

## Data Availability

The data presented in this study are available on request from the corresponding author.
